# Randomized Trial of Marine n-3 Polyunsaturated Fatty Acids for the Prevention of Cerebral Small Vessel Disease and Inflammation in Aging (PUFA Trial): Rationale, Design and Baseline Results

**DOI:** 10.3390/nu11040735

**Published:** 2019-03-29

**Authors:** Gene L. Bowman, Lisa C. Silbert, Hiroko H. Dodge, David Lahna, Kirsten Hagen, Charles F. Murchison, Diane Howieson, Jeffrey Kaye, Joseph F. Quinn, Lynne Shinto

**Affiliations:** 1 Interventional Studies in Aging Center, Hinda and Arthur Marcus Institute for Aging Research, Hebrew SeniorLife, Boston, MA 02131, USA; 2 Department of Medicine, Beth Israel Deaconess Medical Center and Harvard Medical School, Boston, MA 02115, USA; 3 Department of Neurology, Oregon Health and Science University, Portland, OR 97219, USA; silbertl@ohsu.edu (L.C.S.); dodgeh@ohsu.edu (H.H.D.); lahnad@ohsu.edu (D.L.); hagenk@ohsu.edu (K.H.); cfmurch@uab.edu (C.F.M.); dbhowieson@comcast.net (D.H.); kaye@ohsu.edu (J.K.); quinnj@ohsu.edu (J.F.Q.); shintol@ohsu.edu (L.S.); 4 Department of Neurology, Veterans Affairs Portland Health Care System, Portland, OR 97219, USA; 5 Department of Neurology, University of Michigan, Ann Arbor, MI 48109, USA; 6 Department of Biostatistics, University of Alabama at Birmingham, Birmingham, AL 35294, USA

**Keywords:** white matter hyperintensities, cognitive decline, vascular cognitive impairment, executive function, eicosapentaenoic acid, docosahexaenoic acid, neuroimaging, MRI, elderly

## Abstract

Vascular risk factors for age-related cognitive decline are significant, and their management may ultimately prove the most successful strategy for reducing risk and sustaining cognitive health. This randomized, double-blinded, placebo-controlled trial with parallel group allocation to either marine n-3 polyunsaturated fatty acids (n-3 PUFA) or soybean oil placebo assesses the effects on the total volume of accumulation in cerebral white matter hyperintensities (WMH), a potentially modifiable neurovascular component of age-related cognitive decline. Total WMH accumulation over 3 years is the primary endpoint. The safety and efficacy of n-3 PUFA is evaluated in older adults with significant WMH and suboptimum plasma n-3 PUFA as inclusion criteria. One hundred and two non-demented older adults were enrolled with a mean age of 81.1 (±4.4) years, WMH of 19.4 (±16.1) cm^3^, and a plasma n-3 PUFA of 86.64 (±29.21) µg/mL. 61% were female, 28% were apolipoprotein E epsilon 4 carriers, and the mean mini-mental state exam (MMSE) was 27.9 (±1.7). This trial provides an initial evaluation of n-3 PUFA effects on WMH, a reproducible and valid risk biomarker for cognitive decline, as well as on inflammatory biomarkers thought to play a role in WMH accumulation. We present the baseline results and operational experience of enriching a study population on advanced age, blood n-3 PUFA, and magnetic resonance imaging (MRI) derived WMH with biomarker outcomes (WMH, inflammation markers) in a dementia prevention paradigm.

## 1. Introduction

The vascular contributions to dementia, including Alzheimer disease (AD), are significant [1,2] and the reduction in “vascular risk” may significantly reduce the global prevalence and incidence of age-related dementia [3,4]. While AD prevention efforts continue to emphasize targets along the amyloid cascade [5], reducing vascular risk may ultimately prove the most effective approach in reducing age-related cognitive decline and dementia incidence in the near term. However, the paradigm of clinical trials in dementia prevention needs to incorporate reproducible and valid measures of these vascular risk factors that are also relevant to brain health to facilitate the testing of experimental interventions more efficiently. Dementia prevention studies are a major challenge in the general population, requiring the enrollment of thousands of subjects for studies lasting several years [6,7]. One pragmatic approach is to reduce the potential number of subjects needed and the duration of the study by utilizing and validating appropriate surrogate markers of disease. WMH volume is an important cerebrovascular marker related to age-related cognitive decline and all-cause dementia [1,8,9], and appears sensitive to interventions that reduce vascular risk factors [10]. However, this MRI-derived biomarker has not yet been evaluated as a primary outcome measure in the field. This trial provides an initial evaluation of WMH as a surrogate biomarker of disease risk in a non-demented population of older adults.

Higher intakes of fish and plasma n-3 PUFA are associated with reduced WMH volume [11,12,13,14], suggesting that n-3 PUFA effects are more confined to the neurovascular pathology mediators of age-related cognitive decline rather than the underlying mechanisms that govern total brain atrophy in late life specifically (i.e., Alzheimer type pathology). This hypothesis is biologically plausible and supported further by a body of research highlighting several mechanisms, including anti-thrombotic, anti-inflammatory, anti-atherosclerotic/arteriosclerotic plaque forming, and promotion of endothelial cell function reducing cardiovascular disease (CVD) incidence [15]. Several experimental studies demonstrate the down-regulation of inflammatory and atherogenic molecular pathways with n-3 PUFA intake [16,17]. More recent studies suggest that metabolites of eicosapentaenoic acid (EPA) and docosahexaenoic acid (DHA) may directly modulate inflammatory homeostasis, preventing cellular damage and offering neuroprotection [18]. The current trial collects blood appropriately to examine whether these metabolites mediate any effects of n-3 PUFA on the primary endpoints of WMH and inflammatory biomarker changes.

One inflammatory protein of particular interest is the soluble form of intercellular adhesion molecule-1 (ICAM-1), or CD54 (Cluster of Differentiation 54). This nearly ubiquitous transmembrane glycoprotein plays a significant role in the leukocyte migration and its activation [19,20]. The principal binding partners of ICAM-1 are the leukocyte integrin’s lymphocyte function-associated antigen 1 (LFA-1) and macrophage-1 antigen (Mac-1) [21,22]. At sites of inflammation, there is upregulation of ICAM-1 on the endothelial and epithelial cell surface that mediates the adhesion and paracellular migration of leukocytes with activation of LFA-1 receptors [23]. ICAM-1 ligation prolongs antigen presentation by dendritic cells and promotes T cell proliferation and cytokine release [24]. Some evidence suggests that the pathogenesis of cerebral small vessel disease involves the upregulation of sICAM-1 [25]. Some studies, but not all, have demonstrated an association between ICAM-1 and WMH progression [26,27,28,29,30]. More recent evidence suggests that upregulation of sICAM-1 has a role in blood–brain barrier breakdown in older adults which places them at risk for neuroinflammation and cognitive decline [31]. Serial plasma levels of sICAM-1 enables the determination of the effects of n-3 PUFA supplementation and whether its expression appears to mediate the effects of n-3 PUFA on WMH.

Randomized controlled trials of traditional vascular risk factors (e.g., blood pressure, cholesterol, diabetes) are unethical when placebo participants are denied a standard of care. In contrast, a randomized trial using the n-3 PUFA is reasonable because this practice is currently outside the standard of care for cerebral small vessel disease. There are several plausible explanations in support of the notion that n-3 PUFA effects are more confined to neurovascular mechanisms leading to neuroprotection. These include well-established effects on vascular health, leading to recommendations for a standard of care in primary and secondary prevention of coronary heart disease [32], refined further based on more recent clinical trial data [15,33]. On the other hand, n-3 PUFA trials in people with AD which select subjects with low vascular risk factors as a strategy for a more pure AD experiment have not seen efficacy [34,35]. Both observational and experimental studies have shown that the beneficial effects of n-3 PUFA may be limited to the non-carriers of the apolipoprotein E epsilon 4 (*APOE4*) allele [35,36]. Since *APOE4* carriers are more prone to cerebral amyloid than non-carriers [37,38,39], the observation that n-3 PUFA may be more effective in non-carriers suggests that they operate via an amyloid-independent mechanism. The current trial explores this possibility by examining the interaction of treatment by *APOE4* status in a planned secondary analysis.

Our study population focuses on the oldest-old, which are the fastest growing segment of the American population that are at the highest risk for cognitive impairment, significant WMH burden and suboptimal n-3 PUFA status [8,9,40]. The neuropathology in subjects that succumb to dementia at this age is also less dominated by beta-amyloid plaques, neurofibrillary tangles (compared to “young, old” subjects), and *APOE4* genotype [41,42]. Despite the importance of this population in planning for future public health efforts to prevent late-life dementia, these individuals are rarely included in clinical research. This trial focused on people of advanced age for targeting a neurovascular mechanism underlying dementia risk with n-3 PUFA.

**Figure 1:**
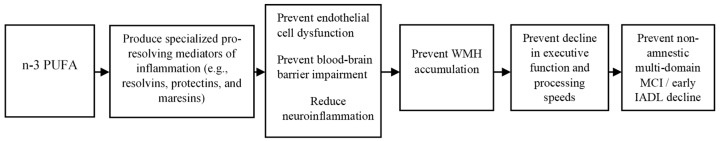
Hypothetical model for n-3 PUFA effects on age-related cognitive decline. Administration of n-3 PUFA makes them available for incorporation into cellular membranes and subsequent release by intracellular phospholipases that convert them into a variety of lipid bioactives. Unesterified circulating n-3 PUFA can arrive at sites of inflammation and undergo conversion into specialized pro-resolving lipid mediators, including the E-series resolvins derived from EPA via p450 metabolism, or aspirin acetylated cyclooxygenase and the D series resolvins, protectins, and maresins, derived from DHA via lipoxygenase or aspirin acetylated COX-2 [49]. Administration of n-3 PUFA is associated with down-regulation of cell adhesion molecules [48] that may govern blood–brain barrier permeability in older adults [31]. These cell adhesion molecules may play a role in cerebral small vessel disease as reflected by the accumulation magnetic resonance imaging derived white matter hyperintensities. Executive function and speed of processing are domains of cognition impacted by the accumulation of WMH, in contrast to episodic memory recall more influenced by deposits of Alzheimer pathology.

In conclusion, this double-blinded, placebo-controlled trial with parallel groups randomized at the individual level reports baseline results from the targeting of WMH with n-3 PUFA in a non-demented stage with significant WMH burden. The population includes a sample from a large and expanding segment of the population at high-risk for dementia and currently under-represented in prevention trials. Omega-3 PUFA have operate on vascular and inflammatory mechanisms which may explain their association with less ischemic strokes [43,44,45], recurrent ventricular arrhythmias [46], congestive heart failure [45], and overall CVD risk [15], which may all reduce risk for age-related dementia. Lower blood concentrations of n-3 PUFA is associated with higher total WMH [11,12], worse executive function [12], and more executive function decline over time [47]. Omega-3 PUFA supplementation may regulate endothelial cell function [48], and there is emerging evidence indicating that lowering cell adhesion molecules binding to endothelial cells may prevent blood–brain barrier breakdown and slow WMH accumulation-mediated decline in cognitive flexibility and processing speeds. Figure 1 summarizes the hypothetical model for how n-3 PUFA prevents age-related cognitive decline.

**Figure 2:**
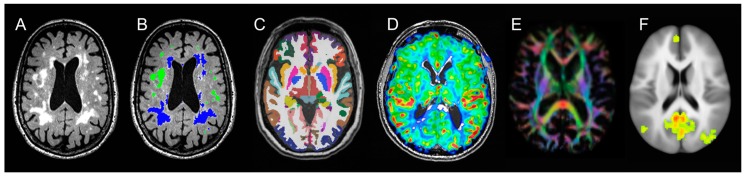
Neuroimaging endpoints available in the PUFA Trial. (**A**). Fluid-attenuated inversion recovery (FLAIR) sequence illustrating prominent total WMH; (**B**). WMH segmentation classified into deep (green) and periventricular (blue); (**C**). T1 grey matter segmentation including medial temporal lobe (brown); (**D**). 2D Pseudo-Continuous Arterial Spin Labeling (pCASL) sequence for cerebral blood flow; (**E**). Diffusion Tensor Imaging (DTI) derived fractional anisotropy direction color-coded; (**F**). fMRI derived default mode network activity at resting state.

The primary aim of this study is to test the hypothesis that a 3-year intervention with marine n-3 PUFA slows the total WMH accumulation in older non-demented elders (75+) versus placebo (Figure 2). This aim will also examine the effects of n-3 PUFA on medial temporal lobe atrophy and regional fractional anisotropy in white matter tracts using diffusion tensor imaging. The secondary aims will test the hypothesis that n-3 PUFA supplementation down regulates blood-based biomarkers of inflammation, increases n-3 PUFA metabolites, and interaction by APOE genotype. The tertiary aim explores the 3 year effects on psychometric and other functional indices historically sensitive to WMH accumulation.

## 2. Materials and Methods

### 2.1. Study Design

This double-blinded, placebo-controlled trial with randomization at the individual level was conducted at Oregon Health and Science University to examine the differences in WMH accumulation trajectories across groups as the primary outcome. Serial plasma samples were collected prospectively over 8 time points (baseline, 3, 6, 12, 18, 24, 30, and 36 months) to confirm compliance, and examine biological activity and pharmacodynamics of the n-3 PUFA supplementation. Neuropsychological tests were collected annually to emphasize domains associated with WMH accumulation. Tests of global cognition and daily functional capacity were collected to determine any significant effects of the intervention on functionally relevant outcomes although we recognize that cognitive and functional change in this relatively healthy population is very modest in this time frame. According to preliminary data obtained from the Oregon Brain Aging Study (OBAS), 50 subjects are needed per arm to detect a 40% reduction in WMH accumulation over the 3 years between groups (80% power; two-tailed alpha = 0.05). Thirty-two subjects are required to detect a 50% reduction in WMH progression over this period (80% power; two-tailed alpha = 0.05).

### 2.2. N-3 PUFA Composition and Dose

The active intervention is a standardized n-3 PUFA formulation (fish oil extract) vs. a standardized placebo soybean oil administered daily over 3 years. The PUFA formulation and dosage has an excellent safety profile and predictable blood concentrations in preliminary studies. Each 9.45 mm in diameter and 23.5 mm long soft gel (ProOmega 3, Nordic Naturals), weighing 1423 mg, contains 1 g of marine lipid concentrate yielding EPA (325 mg) and DHA (225 mg). Nordic Naturals supplied the placebo soft gel containing soybean oil matched for taste, appearance, and smell. Three soft gels offer a daily dose of 1.65 g of marine n-3 PUFA.

### 2.3. Study Population

The PUFA trial enrolled non-demented elders age 75 and older with a Clinical Dementia Rating (CDR) ≤ 0.5 and a mini-mental state exam (MMSE) ≥ 24 with MRI derived total WMH ≥ 5 cm^3^ and blood n-3 PUFA (EPA + DHA < 110 ug/mL or < 5.5 wt%). Eligibility requires the absence of significant depressive symptoms defined as a geriatric depression scale–15 (GDS) of less than 6, with sufficient vision and hearing acuity to complete the full test regimen. An informant with frequent contact, defined as 1 h/day or 1 day/week, was required to verify functional status and the CDR rating.

Potential participants were ineligible if they had a dementing illness (e.g., diagnosis of a neurological disease that might impact cognition, normal pressure hydrocephalus, or Parkinson’s disease) or significant CNS diseases, such as a brain tumor, seizure disorder, subdural hematoma, or cranial arteritis. Significant DSM-IV defined alcohol or substance abuse within the last 2 years, major depression, schizophrenia, or other major psychiatric disorders were excluded. Abnormal laboratory results indicating vitamin B12 deficiency, thyroid disease, or UTI were excluded. Any unstable or significantly symptomatic CVD (e.g., CAD with frequent angina, CHF with dyspnea at rest), uncontrolled hypertension (e.g., >150/90), or clinical symptomatic orthostatic hypotension; diabetes mellitus that requires insulin injections; a history of cortical stroke and cancer within the last 5 years, with the exception of localized prostate cancer (Gleason Grade < 3) and non-metastatic skin cancers; melanomas were excluded. Illnesses that requires >1 visit a month to a clinician and any contraindications to MRI (i.e., heart pacemaker, metal plates or objects in the head, claustrophobia) met exclusion criteria as well.

A screening plasma n-3 PUFA > 5.5 weight percent of total fatty acids were excluded. If potential participants indicated regular supplementation with fish oil on their phone screen, a 4-month washout was permitted and confirmed by blood testing before initial study randomization. Over the counter supplements were not by themselves exclusionary; however, subjects were asked not to change the dosing regimen throughout the trial unless medically indicated and the presence and dose of these agents were known. Potential participants using CNS active medications that had not been on stable doses for at least 2 months (e.g., cimetidine, beta-blockers, and SSRIs) were excluded. Neuroleptics, antiparkinsonian agents, systemic corticosteroids, and narcotic analgesics used for a self-limited time must have been discounted for a period of five half-lives before baseline visit. Cholinesterase inhibitors (i.e., Aricept) and investigational drugs within five half-lives prior to baseline and anticoagulation therapies, including: vitamin K antagonist (warfarin (Coumadin, jantoven), Factor Xa inhibitors: rivaroxaban (Xarelto), fondaparinux (Arixtra), dibigatran (Pradaxa), apixaban (eliquis) and low molecular weight heparins (dalteparin, fragmin), and enoxaparin (Lovenox) were exclusion criteria. Incident use of anticoagulant therapies excluded further study drug allocation. However, participants continue to be followed otherwise.

### 2.4. Outcome Measures

#### 2.4.1. Neuroimaging

MRI data was acquired using a Siemens 3T MRI instrument with phased array RF coils and 32 channel head coils housed in the OHSU advanced imaging research center (AIRC). Subjects underwent anatomical imaging sequences similar to those used in the Alzheimer disease neuroimaging initiative [44]. Full sequence parameters are described in Table 1 and illustrated in Figure 2. Total MRI acquisition time was one hour, 10 min.

MRI-derived WMH was quantified using a customized segmentation algorithm [50]. Briefly, T1 images were segmented into white matter, grey matter (GM), and cerebral spinal fluid (CSF) using Freesurfer (v5.1). The FLAIR image was linearly co-registered to the T1 image, and the average FLAIR signal intensity in the WM was calculated. Clusters of at least 3 voxels in size and of at least 2.5 standard deviations above the mean WM FLAIR signal intensity were used as seeds for a custom cluster growing algorithm. The mean signal intensity of each cluster was calculated and compared to all surrounding voxels. Adjacent voxels of at least 95% of the cluster mean signal intensity were added to the cluster. This process was repeated iteratively until the cluster mean intensity fell below 2-standard deviations above the mean, or until no voxels met the threshold. WMH clusters adjacent to the ventricles were labeled periventricular, all other WMH clusters were labeled as deep, and the combination was labeled total WMH. The final WMH mask was visually inspected and manually corrected before final volume calculation.

Brain MRI secondary outcomes include: (1) voxel-based morphometry (VBM) analysis using FSL software to segment GM in T1 images and calculate GM density in standard Montreal neurological institute (MNI) stereotactic space was used to examine regional differences between groups for the grey matter volumes; (2) Volumetric brain MRI using Freesurfer will be used to determine the group differences in medial temporal lobe volume changes, which is another region that may be sensitive to PUFAs [51,52,53]. The WMH masks will be used to correct the Freesurfer segmentation to provide accurate tissue type volumes because WMH appears iso-intense with GM in T1 sequences and is therefore susceptible to misclassification; (3) Diffusion tensor imaging was used to examine regional and between-group differences in white matter tracts previously linked to PUFA supplementation using TBSS [54]; (4) Pseudo-continuous arterial spin labeling (pCASL) was used to quantify cerebral blood flow and examine regional perfusion differences between groups; (5) Resting-state functional MRI examined connectivity patterns within the default mode network (Table 1 and Figure 2).

#### 2.4.2. Blood-Based Biomarkers

Plasma fatty acids were measured using ultra-performance liquid chromatography-tandem mass spectrometry; inflammatory biomarkers (sICAM-1, sVCAM-1, E-Selectin, TNFα, IL-6, hsCRP), and beta-amyloid metabolites (abeta 40 and 42) by ELISA; lipids by enzymatic methods; and *APOE4* genotyping by PCR [12,55].

Plasma n-3 PUFA ≤ 5.5% of EPA plus DHA of total fatty acids at screening visit 1 used the Holman Omega-3 blood spot test that allows a 10-day turn-around of the results to accelerate the determination of baseline inclusion/exclusion criteria [55,56]. These measures (except APOE4) are collected at baseline, 3, 6, 12, 18, 24, 30, and 36 months. The food frequency questionnaire was collected at baseline and at month 36 to assess the stability of the diet during the study and assess any dietary drift within treatment groups [57].

#### 2.4.3. Neuropsychological Test Battery

The full battery requires about forty-five minutes to administer, and includes: trail making test [58]; WAIS-IV Coding [59]; Category fluency (animals and vegetables); Verbal letter frequency (C and L); Multilingual naming test (MINT) [60]; Craft story 21 recall (immediate and delayed) [61]; Digit spans (forward and backward); Benson complex figure copy (immediate and delayed); Clinical dementia rating [62]; MMSE [63]; and the Montreal cognitive assessment (MoCA) [64]. Administration was as follows: WAIS-IV Coding, MoCA, Craft story 21 immediate recall, benson complex figure copy immediate recall, number span forward, number span backward, category fluency, trail making test A, trail making test B, craft story 21 delayed recall, benson complex figure copy delayed recall, MiNT, and verbal letter fluency. CDR and MMSE were administered annually and independent from the neuropsychological test battery.

#### 2.4.4. Diet and Other Measures

A food frequency questionnaire was collected at baseline and at month 36 to assess the stability of diet during the study [57] and identify dietary drift within the treatment groups. The widely used and brief geriatric depression scale-15 used for screening depression in the elderly is non-somatically focused and can be either observed or self-administered. The scores range from 0–15, with higher scores indicating a more severe depression of mood [65]. Functional measures of a participant’s ability to carry out tasks that are important for daily living are captured by the administration of the instrumental activities of daily living [66]. The timed gait test is a 10-yard (30-foot) course marked on a straight, unobstructed area where the participants are instructed to walk the 15 feet as quickly as possible without running, then cross a line, turn around, and walk back the 15 feet. The mean time of three trials were recorded in seconds [67].

### 2.5. Recruitment and Randomization

#### 2.5.1. Sources of Participants

Mailers were sent across Oregon and Washington counties near Portland and within Oregon, including Multnomah, Clark, Clackamas, Washington, Columbia, Polk, Marion, and Deschutes counties. Also, the NIA-Layton aging and Alzheimer’s disease center maintains direct contact of its ongoing longitudinal aging studies in over 50 aging centers, retirement communities, and community organizations in the Portland metro area. The research data warehouse at OHSU, in partnership with the Vancouver clinic, provided additional recruitment sites.

Potential participants received an invitational letter from the study team that outlined the rationale for the trial and participation details. Before an in-person screening, potential participants were identified by the investigative group through routine clinical contact or a pre-screening process. Prospective subjects were not demented and known to meet as many of the inclusion/exclusion criteria as possible through this process. These subjects attended the screening in the clinic with a study partner and were asked to bring the medications that they were currently taking. If the study partner was unable to attend the clinic screening, a phone interview with the study partner took place and consent was obtained over the phone. Potential participants were not considered participants until they attended the in-clinic screening and relayed their understanding of the study protocol, corroborated verbally, and through signed informed consent.

#### 2.5.2. Enrollment

This study protocol was approved by the OHSU institutional review board on September 26, 2013 and registered at ClinicalTrials.gov as NCT01953705 on September 30, 2013. Informed consent was obtained from both the study subject and the study partner when they arrived at the clinic before any study-related procedures. Demographics and general medical history were collected, including psychiatric history, family history, and history of allergies. A general physical and neurological exam was performed, including vital measures. A review of pre-study medications over the prior month was reviewed. Confirmation of a non-dementia status was made at the Oregon Alzheimer’s Disease Center clinic, where cases are presented and a consensus diagnosis is met prior to enrollment. The MMSE, CDR, geriatric depression score, screening and safety laboratory, and a plasma EPA and DHA in ug/mL were performed. This information was used to establish trial eligibility. The screening visit 1 lasted approximately 2 h. Screening visits 1 and 2 were sometimes combined, and in this case, the participant was asked to fast overnight prior to the clinic appointment. Randomization occurred after eligibility criteria was reviewed. Those ineligible participants were notified and provided explanation. The total number of screen failures and their justification were recorded, including those due to high plasma PUFA index.

Those meeting trial eligibilities were scheduled for a brain MRI within 56 days of screening visit 1 to determine total WMH eligibility. This visit took approximately 3 h and included the administration of a food frequency questionnaire by a nutritionist, a central blood pressure measurement by a research coordinator, and any missing assessments supervised by the study clinician (e.g., general physical and neurological exam). Participants who were ineligible for the study were notified and provided an explanation. The total number of screen failures and justification recorded included those due to lower total WMH volume.

##### Ethnicity of Study Population

Efforts were made to meet goals for recruitment of Asian and African American participants through community presentations and face to face interviews with members of the African American Dementia and Aging project cohort focused on ethnic-diverse populations.

#### 2.5.3. Randomization

Subjects meeting the inclusion criteria were invited for the baseline visit 3 within 56 days of the screening visit 1 and assigned a sequential number determining group assignment determined by a modified randomized minimization algorithm [68]. An independent statistician generated the randomization sequence and balanced the trial arms on age (5-year categories), gender, level of education (up to high school graduate, at least some college, and baccalaureate graduate/post-graduate), WMH volume, and CDR scores.

#### 2.5.4. Blinding

The participants and study staff (study investigators, research associates, and study coordinators) were blind to treatment assignment. Data analysis was performed by the primary study statistician who was blinded to the treatment status. The secondary project biostatistician that created the randomization scheme ensured the blinding of all study medications and data records. Study supplementation was received, processed, and managed (handled, labeled, and distributed) by pharmacists in research pharmacy services at OHSU who were otherwise independent of the study. The effectiveness of the study blinding was assessed with a short questionnaire completed by the study evaluators, subjects, and investigators recording their knowledge of the group assignments. The randomization code was not broken without consent from the DSMB under unusual circumstances, such as in the event of numerous serious adverse events before the end of the study.

### 2.6. Assessment of Compliance

Pill counts measured compliance. In addition to the in-clinic checks, each subject received a phone call to check on compliance on a 3-month cycle. If compliance dropped below 80%, they were contacted by phone to find a solution and to aid in ways to maintain compliance with the protocol. Plasma fatty acid measurements assessed formal compliance after completion of the trial to corroborate the self-report pill counts.

### 2.7. Analytical Approach and Statistical Power

#### 2.7.1. Analytical Approach

First, a univariate analysis using changes in WMH accumulation (Aim 1), sICAM-1 (Aim 2), and cognitive test scores (Aim 3) between baseline and three follow-up assessments (baseline, 12, 24, and 36 months) for WMH, sICAM-1, and cognitive outcomes with a predictor variable being active vs. placebo. The linear mixed effects multiple regression models fit each aim endpoint and the main predictor as a dummy variable for PUFA active vs. PUFA placebo as a reference and its interaction with a time variable. Control variables included age at baseline, sex, history, and incidence of vascular diseases, including hypertension, APOE4 allele, and depression. Intercept and time variables (years from baseline) blocked within subjects were treated as random effects in all models. We used an unstructured error covariance structure and estimated parameters using restricted maximum likelihood procedures. Missing data points in the analytical sample were considered missing at random, and the above approach is adequate under this assumption [69]. An interaction term between the active and placebo groups and time is of our predictor of substantive interest. That is, we are interested in examining the magnitude and significance of the difference in time slopes between the active and placebo groups. In all models, quadratic and higher order time variables will be included if it improves the model fitness. The overall fit of the models was examined using a combination of formal fit criteria and visual inspection of residual plots. Results were considered significant if *p* < 0.05. The significance level for subgroup analysis was set at 0.05, but we provided Bonferroni-adjusted *p*-values to correct for multiple statistical tests.

##### Modified Intention-To-Treat Analysis

Student’s t-tests will provide descriptive analysis based on ITT. This analysis was modified to include all subjects randomized to treatments with at least one follow-up primary outcome measure to estimate change (e.g., MRI derived WMH).

##### Per-Protocol Analysis

Participants meeting the pre-specified inclusion criteria, including: compliance to the n-3 PUFA treatment represented by pill counts at 80% or greater (or blood plasma n-3 PUFA > 110 umol/L), having at least 1 follow-up outcome measure, and without other protocol violations. Differences in those that adhere to the protocol versus those that do not in relation to the primary endpoints are evaluated.

#### 2.7.2. Statistical Power

##### Sample Size Calculations

Attenuated rate of total WMH progression: The sample size calculation is based upon the information obtained from the Oregon brain aging study (OBAS), demonstrating that the oldest-old adults with CDR ≤ 0.5 accumulated a mean total WMH volume of 7.0 cc over 3 years. Assuming a 50% reduction in WMH accumulation over 3 years among the active group (mean change (SD), among the placebo group: 7.0 ± 5, mean change (SD), and among the n-3 PUFA group: 3.5 ± 5), we would have 80% power to detect this difference with a sample size of 33 subjects per arm at alpha = 0.05 (two-tailed). Thus, with a conservative estimate of 30% attrition over 3 years, we will achieve at least 80% power to detect a reasonable effect size (50%) with a sample size of 100 subjects at baseline with 70 completers.

Slowed rate of plasma soluble ICAM-1 increase: Our preliminary data demonstrates that the mean plasma sICAM-1 in non-demented participants of the OBAS under the age of 80 and those aged 80 and older was 219.19 ng/mL (± 39.12) and 269.34 ng/mL (±78.89), respectively. We expect in our trial population, age 75 and older, that a reduction of plasma sICAM-1 in the active group will achieve plasma PUFA levels of that observed in the younger participants of OBAS (< 80 years old). Under this scenario, we would have over 93% power to detect a reduction in plasma sICAM-1 of this magnitude with the proposed sample (*n* = 35) for each arm (i.e., about 35 each at the end of the 3rd year assessment and after 30% attrition with 100 enrolled at baseline).

Slowed rate of trails B and digit symbol change: This exploratory aim assumes an annual rate of change in trail making test-B of 4 s and a digit symbol score of 0.75 in older adults (80 and older in the Oregon brain aging study) and a total 3-year change of 12 (±13) and 2.25 (±2), respectively. With 35 participants in each arm at the end of 3 years follow-up, we would have 80% power to detect the standardized effect size of 0.7 (e.g., trail B change scores: 12 (placebo) vs. 3.7 (active), digit symbol: 2.3 (placebo) vs. 0.7 (active). The distribution of changes in neuropsychological test scores obtained in this trial provide the basis for a sufficiently powered trial in the future.

### 2.8. Trial Monitoring

Adverse events (AE) were monitored in “real time” with weekly review of AEs, with study staff using clinical judgment to determine whether to report to IRB. A data safety monitoring board was assembled and convened bi-annually to receive updates on study progress and review the unblinded study data, including any AE coded by organ systems. Assessment of AE also included solicited non-specific changes (e.g., “do you feel different since beginning the treatment?”). The 12-item AE checklist covers all major organ systems, the nature of each AE, its severity (mild, moderate, or severe), its likely relationship to study treatment (definite, probable, possible, not related, or unknown), its duration, and any necessary treatment modifications or adjustments. In addition to the AE monitoring and recording, laboratory assessments of basic metabolic and liver function, complete blood counts, and prothrombin time/INR were performed at screening and at months 6, 1,2 and then annually. Participants were reminded and encouraged during clinic visits and phone checks to contact the study coordinator or investigative team if any moderate or serious AE had occurred.

## 3. Results

**Figure 3:**
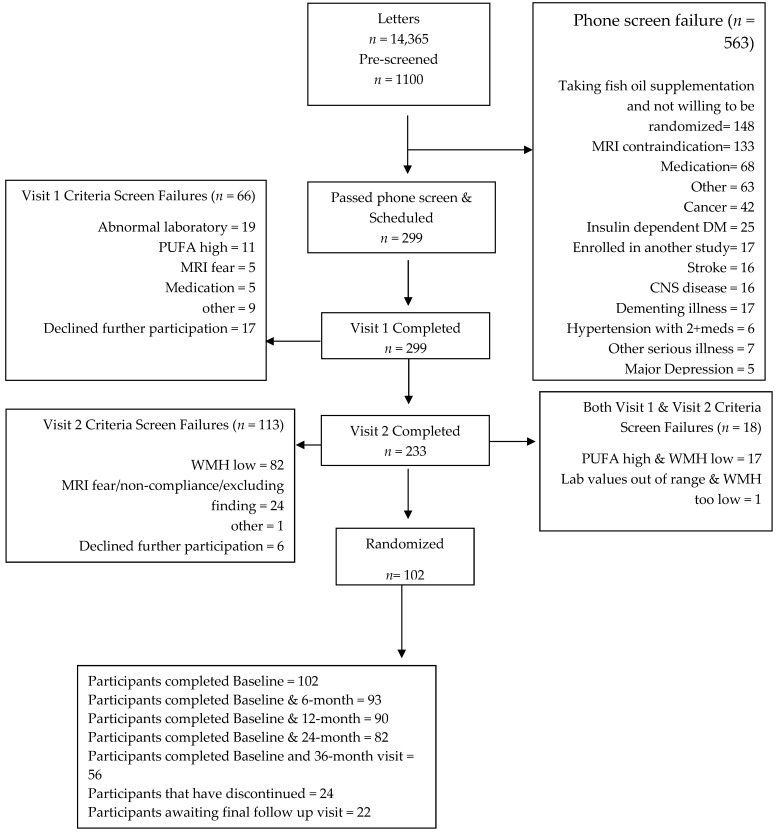
PUFA Trial Participant Flow.

NIH/NIA funding was received in September 2013. Enrollment was initiated in May of 2014 and concluded in June of 2016. The last follow-up for the last participant is scheduled for August 30, 2019. Figure 3 illustrates the study flow where recruitment was initiated, with mass mailers sent to the surrounding communities. The mass mailers included the rationale and objective, and a description of the study visits with general eligibility criteria. It was mailed to over 14,000 residential addresses targeting people that have visited OHSU and met partial inclusion criteria, identified via cohort discovery medical record software. Potential participants were then phone screened (*n* = 1100), yielding 299 older adults meeting initial inclusion criteria, and were invited into the clinic for additional screening with brain MRI, blood work, physical and neurological examinations, and neuropsychological testing. There were 233 brain MRI scans performed at the screening, of which 108 (46.35%) met the inclusion criteria. These subjects were then screened for blood n-3 PUFA after an overnight fast. Ninety-two percent (216/233) met blood n-3 PUFA eligibility using blood spot criteria. Eighty-one participants met initial plasma n-3 PUFA (EPA+DHA) < 100 ug/mL criteria. Thirteen percent (148/1100) of older adults who expressed interest in the study reported that they were already taking fish oils and unwilling to stop administration for a washout period to participate. These interested older adults were excluded from participation through the phone screen. One-hundred and two subjects were enrolled, and 24 have discontinued as of March 5, 2019.

The randomization was successful with treatment groups balanced on age, gender, education, CDR, and MRI derived WMH (Table 2). The mean baseline age was 80.8 years, and 61% were female. The mean plasma n-3 PUFA was 85.64 ug/mL, with a plasma EPA of 22.3 and a plasma DHA of 63.1 ug/mL. The MRI methods are presented in Table 1. The mean total brain volume was 882.0 cm^3^ and the mean total WMH was 19.4 cm^3^. All participants had a clinical dementia rating ≤ 0.5 and MMSE ≥ 24. The prevalence of CDR of 0 was 70%. The mean MMSE was 27, trail making test part B completion speed was 118 s, and digit symbol was 48.

## 4. Discussion and Conclusions

This randomized controlled trial enrolled study subjects by nutritional status and neuroimaging parameters. It includes the enrollment of subjects age 75 and older with representative blood n-3 PUFAs and significant MRI-derived WMH burden, reflecting the neurovascular pathology being targeted by the n-3 PUFA intervention. Excluding those with high n-3 PUFA may improve the opportunity to observe biological effects that would otherwise already be maximized in those with optimum n-3 PUFA. The remarkable safety profile of the intervention is notable, particularly in this oldest-old population with documented silent cerebrovascular disease. The targeting of cerebral WMH volume as the primary outcome and surrogate biomarker of disease risk in older adults is a unique approach to demonstrate target engagement in a dementia prevention paradigm. WMH is evaluated independently of the intervention results to provide valuable insights into its utility for future trials. The blood spot n-3 index performance is benchmarked against standard blood plasma n-3 PUFA quantification to determine its feasibility for allowing a more rapid enrollment process that includes people with ‘nutritional risk’ for cognitive decline.

The MMSE scores in the current study (mean 27.9) are similar to the US population-based norms according to age and education demonstrating that elders, age 70–75 years, with a mean MMSE of 28, and mean MMSE of 27 for those aged 80–84 years [70]. The MoCA scores in the current study (mean 24.4) are also in line with population norms in elders age 70–80 with > 12 years of education (mean 23.6, median 24) [71]. Trail making test part A (mean 39.1) and B (mean 118.7) completion times in the current study are also comparable to reports in the literature for those 75–79 years old with > 12 years education (mean TMT-A of 41.7 and trail B of 100.6), 80–84 years old (mean TMT-A of 55.3 and trails B of 132.1), and 85-89 years old (mean TMT-A of 57.5 and trails B of 167.6) [72]. These data suggest that cognitive performance in the current study represents that of other non-demented oldest-old populations in the US.

The mean total plasma n-3 PUFA (EPA+DHA) in the current study was 85.6 ug/mL (EPA mean of 22.4; mean DHA of 63.2 ug/mL). In other reports in non-demented older adults with a mean age of 69, the mean total plasma EPA was 24 umol/L (21.2 ug/mL) and DHA mean 78.6 umol/L (69.5 ug/mL) [73] and in the oldest-old with a mean age of 87, a mean total plasma EPA of 15.6 ug/mL and DHA of 67.3 ug/mL [12]. In another clinical trial in non-demented adults with the median age of 64 years, the baseline mean total serum EPA was 26.1 ug/mL [15]. In another study of younger men and women (mean age of 50 years old), the median plasma phospholipid n-3 PUFA (EPA+DHA) was 67.6 ug/mL (EPA of 11.1 and DHA of 41.8 ug/mL) [74]. Together, these data suggest that the baseline n-3 PUFA status in the current study is comparable to that reported in similar populations, but certainly not lower status, even with operational efforts in place to exclude high n-3 PUFA (total plasma EPA+DHA ≥ 110 ug/mL).

The volumetric MRI measures for whole brain, intracranial, and hippocampal volumes in the current study are similar to those reported by others in dementia-free, elderly populations [8,75,76]. Although direct comparisons are often difficult due to the differences in volumetric segmentation methodologies, the total burden of WMH observed in this study is higher than that reported previously in population-based studies of older individuals, which have ranged from 1.8–16.38 cm^3^ [8,77,78,79]. The increased WMH burden in the current study cohort is likely a reflection of the study design, which excluded individuals with little to no total WMH, with the intended target population being those at highest risk for WMH progression and subsequent vascular cognitive impairment.

The cognitive performance, plasma n-3 PUFA, and neuroimaging results are consistent with other reports that include these characteristics in older adults in the US. This study includes a non-demented population enriched on advanced age, suboptimum total plasma n-3 PUFA, and significant cerebrovascular disease (i.e., high WMH burden). The outcome will inform the next steps in targeting the vascular components of age-related cognitive decline with nutritional interventions.

## Figures and Tables

**Table 1:** MRI Sequences Deployed in the PUFA Trial.

Sequence	Orientation	Number of Volumes	Acquisition Matrix	Slice Thickness (mm)	# of Slices	TR (ms)	TE (ms)	TI (ms)	Flip Angle	In Plane Resolution (mm)
T1 MPRAGE	Axial	1	256 × 192	1	144	2300	3.45	1200	12°	1 × 1
FLAIR	3D	1	512 × 512	1	160	6000	388	2100	120°	0.488 × 0.488
PCASL	Axial	1 M0, 252	64 × 64	4	10	3500	13	-	90°	3 × 3
DTI	Axial	30 dirs., 6B0, 2avg	128 × 128	2	72	9100	88	-	90°	2 × 2
Diffusion Field map	Axial	1	120 × 120	2	72	790	5.19	-	60°	2 × 2
T2	Axial	1	256 × 256	4	28	693	20	-	20°	0.859 × 0.859
PD—T2 TSE	Axial	2	256 × 232	3	48	3000	11	-	150°	0.938 × 0.938
Resting state fMRI	Axial	170	128 × 128	4	30	2020	38	-	80°	1.875 × 1.875

**Table 2:** Baseline Characteristics of the Participants and According to Randomization (*n* = 102).

	N	Total	A	B	*p* for Diff
Age, years; mean (SD), min-max	102	81.1 (4.4) 75–96	81.1 (4.4), 75–93	81.2 (4.4), 75–96	0.92
Female, *n* (%)	102	62 (60.8%)	31 (60.8%)	31 (60.8%)	1.00
Race, *n* (%)					
White	102	98 (96.1%)	51 (100%)	47 (92.2%)	0.12
Education, *n* (%)					
High school graduate or less	102	24 (23.5%)	10 (19.6%)	14 (27.5%)	0.48
Some college	102	20 (19.6%)	11 (21.6%)	9 (17.6%)	0.80
College graduate or advanced degree	102	58 (56.9%)	30 (58.8%)	28 (54.9%)	0.84
Body mass index, mean (SD)	102	26.8 (4.3)	26.2 (3.8)	27.4 (4.8)	0.18
Blood pressure, Systolic, mmHg; mean (SD)	102	146.0 (16.3)	145.2 (18.6)	146.7 (13.7)	0.63
Blood pressure, Diastolic, mmHg; mean (SD)	102	69.8 (11.9)	70.7 (13.0)	68.9 (10.7)	0.44
Pulse pressure, mmHg; mean (SD)	102	76.2 (18.3)	74.5 (19.6)	77.8 (16.8)	0.35
Clinical Dementia Rating = 0; *n* (%)	102	71 (69.6%)	36 (70.6%)	35 (68.6%)	1.00
Geriatric Depression Scale; mean (SD)	102	1.5 (1.4)	1.3 (1.3)	1.6 (1.6)	0.22
Instrumental Activities Daily Living; mean (SD)	102	0.1 (0.4)	0.1 (0.2)	0.1 (0.5)	0.59
Gait speed, seconds; mean (SD)	99	16.0 (4.1)	15.5 (3.8)	16.4 (4.4)	0.29
*APOE4* carrier, *n* (%)	102	28 (27.5%)	15 (29.4%)	13 (25.5%)	0.82
**Neuroimaging; mean (SD)**					
MRI, cm^3^					
Intracranial volume, Total	101	1884.1 (165.9)	1874.3 (151.3)	1894.2 (180.6)	0.55
Brain volume, Total	101	882.0 (80.1)	875 (73.1)	889.1 (86.8)	0.38
Ventricular volume, Total	101	49.3 (21.6)	47.7 (17.5)	50.9 (25.2)	0.46
White Matter Hyperintensity volume, Total	101	19.4 (16.1)	18.9 (15.3)	19.9 (17.1)	0.76
Subcortical deep WMH	101	1.9 (1.6)	2.1 (1.8)	1.7 (1.2)	0.16
Periventricular WMH	101	17.4 (16.0)	16.7 (15.2)	18.1 (16.9)	0.66
Hippocampal volume, Total	101	7.5 (0.9)	7.5 (0.9)	7.5 (0.9)	0.74
**Neuropsychological battery, mean (SD)**					
Mini-Mental State Examination	102	27.9 (1.7)	28.2 (1.8)	27.6 (1.7)	0.10
Montreal Cognitive Assessment	102	24.4 (3.1)	24.7 (3.0)	24.0 (3.2)	0.24
WAIS-IV Coding Digit Symbol	100	48.8 (12.4)	51.4 (13.8)	46.3 (10.5)	0.04
Trail Making Test A, seconds	102	39.1 (12.9)	37.6 (12.3)	40.6 (13.4)	0.24
Trail Making Test B, seconds	102	118.7 (63.1)	115.7 (62.2)	121.6 (64.6)	0.64
Craft Story immediate, verbatim	102	18.4 (6.7)	19.1 (7.4)	17.7 (6.0)	0.31
Craft Story immediate, paraphrase	102	13.9 (4.0)	14.1 (4.3)	13.6 (3.8)	0.51
Craft Story delayed, verbatim	102	15.0 (6.3)	15.5 (7.0)	14.5 (5.5)	0.42
Craft Story delayed, paraphrase	102	12.5 (4.2)	12.9 (4.6)	12.0 (3.7)	0.30
Category Fluency, Animals	102	19.0 (4.9)	19.2 (4.7)	18.9 (5.3)	0.77
Category Fluency, Vegetables	102	13.5 (4.2)	13.6 (4.8)	13.5 (3.5)	0.94
Multilingual naming test	102	29.7 (2.2)	29.8 (2.4)	29.5 (2.0)	0.59
**Biochemical and metabolic measures**					
Plasma eicosapentaenoic acid (EPA), ug/mL	101	22.43 (11.49)	23.08 (11.87)	21.78 (11.18)	0.57
Plasma docosahexaenoic acid (DHA), ug/mL	101	63.20 (20.46)	62.59 (18.80)	63.82 (22.21)	0.76
Plasma EPA+DHA, ug/mL	101	85.64 (29.21)	85.67 (28.28)	85.60 (30.41)	0.99
Plasma vitamin B12, pg/mL	101	694.4 (480.9)	743.5 (498.2)	644.4 (462.3)	0.30
Complete blood count					
White cells, K/cu mm; mean (SD)	102	6.5 (1.9)	6.4 (2.1)	6.5 (1.7)	0.81
Red cells, M/cu mm; mean (SD)	102	4.4 (0.5)	4.4 (0.5)	4.5 (0.5)	0.34
Hemoglobin, g/dL; mean (SD)	102	13.6 (1.4)	13.5 (1.4)	13.7 (1.4)	0.54
Hematocrit, %; mean (SD)	102	41.1 (3.9)	40.7 (3.9)	41.4 (3.8)	0.35
Platelet count, K/cu mm; mean (SD)	102	221.5 (54.7)	223.7 (60.2)	219.3 (49.1)	0.68
Complete metabolic panel, mean (SD)					
Sodium, mmol/L	102	138.9 (2.6)	138.6 (2.9)	139.2 (2.3)	0.22
Potassium, mmol/L	102	3.9 (0.3)	3.9 (0.3)	4.0 (0.3)	0.38
Chloride, mmol/L	102	105.0 (3.0)	104.7 (3.3)	105.4 (2.7)	0.23
Carbon Dioxide, mmol/L	102	27.5 (1.9)	27.3 (1.8)	27.7 (1.9)	0.25
Blood Urea Nitrogen, mg/dL	102	18.0 (5.5)	18.0 (5.7)	17.9 (5.4)	0.99
Creatinine, male, mg/dL	40	1.0 (0.2)	1.0 (0.2)	1.0 (0.2)	0.40
Creatinine, female, mg/dL	62	0.8 (0.2)	0.8 (0.2)	0.8 (0.1)	0.66
Glucose, mg/dL	102	98.3 (16.7)	98.6 (15.7)	98.1 (17.7)	0.88
Calcium, mg/dL	102	9.1 (0.4)	9.1 (0.3)	9.1 (0.4)	0.38
Aspartate Aminotransferase Test, U/L	102	23.6 (8.1)	22.5 (5.9)	24.8 (9.7)	0.15
Alanine Aminotransferase Test, U/L	102	25.5 (8.7)	24.1 (5.9)	27.0 (10.7)	0.09
Alkaline Phosphatase, male, U/L	40	74.5 (19.6)	69.7 (17.4)	79.4 (20.8)	0.12
Alkaline Phosphatase, female, U/L	62	82.2 (20.6)	80.9 (20.5)	83.5 (21.0)	0.63
Total Bilirubin, mg/dL	102	0.7 (0.3)	0.6 (0.3)	0.7 (0.3)	0.74
Total Protein, g/dL	102	7.3 (0.4)	7.4 (0.4)	7.3 (0.4)	0.66
Albumin, g/dL	102	3.8 (0.2)	3.8 (0.2)	3.8 (0.2)	0.93
International Normalized Ratio	100	1.0 (0.1)	1.0 (0.1)	1.0 (0.1)	0.28
Thyroid Stimulating Hormone, mIU/L	101	2.2 (1.6)	1.8 (0.9)	2.5 (2.0)	0.03
Uric acid, mmol/L	96	0.33 (0.08)	0.33 (0.09)	0.33 (0.08)	0.89
